# Prediction of survival in patients with IDH-wildtype astrocytic gliomas using dynamic *O*-(2-[^18^F]-fluoroethyl)-l-tyrosine PET

**DOI:** 10.1007/s00259-020-04695-0

**Published:** 2020-02-07

**Authors:** Elena K. Bauer, Gabriele Stoffels, Tobias Blau, Guido Reifenberger, Jörg Felsberg, Jan M. Werner, Philipp Lohmann, Jurij Rosen, Garry Ceccon, Caroline Tscherpel, Marion Rapp, Michael Sabel, Christian P. Filss, Nadim J. Shah, Bernd Neumaier, Gereon R. Fink, Karl-Josef Langen, Norbert Galldiks

**Affiliations:** 1 Department of Neurology, Faculty of Medicine and University Hospital Cologne, University of Cologne, Kerpener St. 62, 50937 Cologne, Germany; 2 Institute of Neuroscience and Medicine (INM-3, -4, -5), Research Centre Juelich, Leo-Brandt-St. 5, 52425 Juelich, Germany; 3 Department of Neuropathology, Faculty of Medicine and University Hospital Cologne, University of Cologne, Cologne, Germany; 4 Present Address: Institute of Neuropathology, University Hospital Essen, Essen, Germany; 5 Institute of Neuropathology, Heinrich Heine University, Duesseldorf, Germany; 6 Center of Integrated Oncology (CIO), University of Duesseldorf, Duesseldorf, Germany; 7 Department of Neurosurgery, Heinrich Heine University, Duesseldorf, Germany; 8 Department of Nuclear Medicine, University Hospital RWTH Aachen, Aachen, Germany; 9 Department of Neurology, University Hospital RWTH Aachen, Aachen, Germany; 10 Center of Integrated Oncology (CIO), University of Aachen, Aachen, Germany; 11 Center of Integrated Oncology (CIO), University of Cologne, Cologne, Germany

**Keywords:** FET PET, High-grade glioma, IDH mutation, MGMT promoter methylation, Overall survival

## Abstract

**Purpose:**

Integrated histomolecular diagnostics of gliomas according to the World Health Organization (WHO) classification of 2016 has refined diagnostic accuracy and prediction of prognosis. This study aimed at exploring the prognostic value of dynamic *O*-(2-[^18^F]-fluoroethyl)-l-tyrosine (FET) PET in newly diagnosed, histomolecularly classified astrocytic gliomas of WHO grades III or IV.

**Methods:**

Before initiation of treatment, dynamic FET PET imaging was performed in patients with newly diagnosed glioblastoma (GBM) and anaplastic astrocytoma (AA). Static FET PET parameters such as maximum and mean tumour/brain ratios (TBR_max/mean_), the metabolic tumour volume (MTV) as well as the dynamic FET PET parameters time-to-peak (TTP) and slope, were obtained. The predictive ability of FET PET parameters was evaluated concerning the progression-free and overall survival (PFS, OS). Using ROC analyses, threshold values for FET PET parameters were obtained. Subsequently, univariate Kaplan-Meier and multivariate Cox regression survival analyses were performed to assess the predictive power of these parameters for survival.

**Results:**

Sixty patients (45 GBM and 15 AA patients) of two university centres were retrospectively identified. Patients with isocitrate dehydrogenase (IDH)-mutant or O^6^-methylguanine-DNA-methyltransferase (MGMT) promoter-methylated tumours had a significantly longer PFS and OS (both *P* < 0.001). Furthermore, ROC analysis of IDH-wildtype glioma patients (*n* = 45) revealed that a TTP > 25 min (AUC, 0.90; sensitivity, 90%; specificity, 87%; *P* < 0.001) was highly prognostic for longer PFS (13 vs. 7 months; *P* = 0.005) and OS (29 vs. 12 months; *P* < 0.001). In contrast, at a lower level of significance, TBR_max_, TBR_mean_, and MTV were only prognostic for longer OS (*P* = 0.004, *P* = 0.038, and *P* = 0.048, respectively). Besides complete resection and a methylated MGMT promoter, TTP remained significant in multivariate survival analysis (all *P* ≤ 0.02), indicating an independent predictor for OS.

**Conclusions:**

Our data suggest that dynamic FET PET allows the identification of patients with longer OS among patients with newly diagnosed IDH-wildtype GBM and AA.

## Introduction

For decades, important general clinical prognostic factors in patients with malignant gliomas including glioblastoma have been patient age, the extent of tumour resection, and the patient’s overall clinical status as evaluated by the Karnofsky Performance Score (KPS) [1–6]. More recently, several molecular markers have additionally gained pivotal attention regarding prognostication, and have consecutively contributed to the revised classification of the World Health Organization (WHO) Classification of Tumours of the Central Nervous System 2016 [7]. Since this revision, the classification integrates histologic features and selected molecular biomarkers, in particular the mutation status of the isocitrate dehydrogenase (IDH) genes 1 or 2 and the 1p/19q co-deletion status. Compared with the previous WHO classification of 2007 that was based on histological features only [8], the inclusion of these biomarkers markedly improved diagnostic accuracy and allowed for a better prediction of the individual prognosis [9, 10]. Importantly, the prognosis of most patients with IDH-wildtype astrocytic gliomas corresponding histologically to WHO grades II or III has been found to be comparable to that of patients with glioblastoma (GBM) of WHO grade IV, suggesting that genotype is prognostically more important than phenotype in these most common group of gliomas [10, 11].

Besides the estimation of patients’ prognosis using clinical or molecular parameters, imaging parameters obtained from amino acid PET seems to be of value for prognostic estimations in newly diagnosed and untreated glioma patients. As suggested by the response assessment in neuro-oncology (RANO) working group [12], amino acid PET can provide relevant additional information in clinically equivocal situations, and, moreover, prognostic information of glioma patients prior to treatment, which may be of great value for patient counselling.

The increased uptake of amino acids such as O-(2-[^18^F]fluoroethyl)-l-tyrosine (FET) by glioma compared with healthy brain parenchyma seems to be caused predominantly by increased transport of large neutral amino acids through the plasma membrane via the l-type amino acid transporter (LAT) system, especially by the subtypes LAT1 and LAT2 [13]. Besides static PET imaging using radiolabelled amino acids [12, 14], the value of dynamic PET imaging using the tracer FET has gained attraction for glioma grading [15, 16], the detection of the most malignant foci within non-enhancing gliomas on MRI [17, 18], the diagnosis of treatment-related changes such as radiation injury or pseudoprogression [19–23], and the assessment of prognosis in newly diagnosed and untreated gliomas. Regarding the latter indication, the value of dynamic FET PET for prognostication has been demonstrated for gliomas characterized by the 2007 WHO classification [24, 25] as well as by the WHO classification of 2016 [18, 26, 27].

In addition to the prognostic importance of the IDH-mutation status for glioma patients, recent PET studies suggest that within patients with molecularly defined gliomas the further identification of patients with an additional survival benefit using dynamic FET PET parameters is possible [18, 26, 27]. Over and above the prognostic benefit of an IDH mutation, the prediction of an additional survival benefit derived from dynamic FET PET parameters has been predominantly observed for patients with IDH-mutant astrocytic gliomas of the WHO grades II and III [18, 26, 27].

To date, studies evaluating the prognostic value of dynamic FET PET in prognostically unfavourable gliomas defined by neuropathological characteristics remain scarce. Thus, we evaluated in a selected patient subgroup with newly diagnosed and prognostically unfavourable IDH-wildtype astrocytic gliomas of WHO grades III and IV the value of static and dynamic FET PET parameters regarding their significance to predict prognosis before treatment initiation.

## Methods

### Patients

We retrospectively assessed data from 60 adult glioma patients (median age, 55 years; age range, 21–78 years; 25 women and 35 men) of two university centres with neuropathologically confirmed newly diagnosed malignant astrocytic glioma (GBM, *n* = 45; anaplastic astrocytoma (AA), *n* = 15) who underwent dynamic FET PET imaging at the Research Centre Juelich, Germany, at initial diagnosis, i.e. before biopsy, resection, or any other kind of treatment. The data had been acquired from 2009 to 2017. Clinical data of all patients, including first-line treatment, are listed in Table 1. The local ethics committees approved the retrospective analysis of the data. There was no conflict with the Declaration of Helsinki. Before PET imaging, all patients had given written informed consent for the PET investigation and the use of the data for scientific purposes.

**Table 1:** Patient characteristics

Characteristic	Number
Patients	60
Gender (m/f)	35/25
Median age; range	55; 21–78 years
Median Karnofsky Performance Score; range	100%; 60–100%
WHO grade III anaplastic astrocytoma	15
IDH mutant	8
IDH wildtype	7
MGMT promoter methylated	12 (80%)
Contrast enhancement	10 (67%)
Positive FET uptake*	12 (80%)
WHO grade IV glioblastoma	45
IDH mutant	7
IDH wildtype	38
MGMT promoter methylated	23 (51%)
Contrast enhancement	44 (98%)
Positive FET uptake*	44 (98%)
Biopsy	21
Partial resection	10
Complete resection	29
First-line therapy following resection/biopsy	
Chemoradiation with temozolomide ^1^	49 (81%)
RT + lomustine-temozolomide ^2^	2 (4%)
RT + bevacizumab ^3^	1 (2%)
Radiotherapy alone	8 (13%)

*f* female, *IDH* isocitrate dehydrogenase, *m* male, *MGMT* O^6^-methylguanine-DNA-methyltransferase, *RT* radiotherapy,

*FET uptake higher than the background activity, visually assessed by two experienced raters

^1^Treatment according to Stupp et al. [40]

^2^Treatment according to Herrlinger et al. [45]

^3^Treatment according to Herrlinger et al. [46]

### FET PET imaging

As described previously, the amino acid FET was produced via nucleophilic ^18^F-fluorination with a radiochemical purity of greater than 98%, specific radioactivity greater than 200 GBq/μmol, and a radiochemical yield of about 60% [28]. According to national and international guidelines for brain tumour imaging using labelled amino acid analogues [29, 30], all patients fasted for at least 4 h before the PET measurements. All patients underwent a dynamic PET scan from 0 to 50 min post-injection of 3 MBq of FET per kg of body weight. PET imaging was performed either on an ECAT Exact HR+PET scanner (*n* = 37 patients) in 3-dimensional mode (Siemens, Erlangen, Germany) (axial field-of-view, 15.5 cm) or simultaneously with 3 T MR imaging using a BrainPET insert (*n* = 23 patients) (Siemens, Erlangen, Germany). The BrainPET is a compact cylinder that fits into the bore of the Magnetom Trio MR scanner (axial field of view, 19.2 cm) [31].

Iterative reconstruction parameters were 16 subsets, 6 iterations using the OSEM algorithm for the ECAT HR+PET scanner and two subsets, and 32 iterations using the OP-OSEM algorithm for the BrainPET. Data were corrected for random, scattered coincidences, dead time, and motion for both systems. Attenuation correction for the ECAT HR+PET scan was based on a transmission scan, and for the BrainPET scan, a template-based approach [31]. The reconstructed dynamic data sets consisted of 16 time frames (5 × 1 min; 5 × 3 min; 6 × 5 min) for both scanners.

To optimize comparability of the results related to the influence of the two different PET scanners, reconstruction parameters, and post-processing steps, a 2.5-mm 3D Gaussian filter was applied to the BrainPET data before further processing, resulting in an image resolution of approximately 4 mm full width at half maximum (FWHM) (image resolution of the ECAT HR+PET scanner, approximately 6-mm FWHM). In phantom experiments using spheres of different sizes to simulate lesions, this filter kernel demonstrated the best comparability between PET data obtained from the ECAT HR+PET and the BrainPET scanner [32].

### FET PET data analysis

FET PET data analysis was performed as described previously [33]. In brief, for the evaluation of FET data, summed PET images over 20–40 min post-injection were used. A larger crescent shaped reference ROI was placed in the semioval centre of the contralateral unaffected hemisphere including white and grey matter [30]. Mean amino acid uptake in the tumour was determined by a 2-dimensional auto-contouring process in the transversal slice containing the voxel with the maximum tumour uptake using a tumour-to-brain ratio (TBR) of 1.6, as described previously [34, 35]. For the calculation of the maximal amino acid uptake, a circular region-of-interest (ROI) with a diameter of 1.6 cm was centred on the voxel with the maximum tumour uptake [33]. Maximum and mean TBRs (TBR_max_, TBR_mean_) were calculated by dividing the mean SUV of the tumour ROIs by the mean SUV of healthy brain tissue. The FET metabolic tumour volume (MTV) was determined by a 3-dimensional auto-contouring process using a TBR of 1.6 or more using the software PMOD (Version 3.9, PMOD Technologies Ltd.).

As described previously [33], time-activity curves (TACs) of FET uptake (mean SUV) in the tumour were generated by the application of a spherical volume-of-interest (VOI) with a volume of 2 mL centred on the voxel with the maximum tumour uptake and the reference ROI as described above to the entire dynamic dataset. A reference TAC was generated by placing a reference ROI in the unaffected brain tissue as reported [33]. For TAC evaluation, the time-to-peak (TTP; time in minutes from the beginning of the dynamic acquisition up to the maximum SUV of the lesion) was determined. In cases with steadily increasing FET uptake without identifiable peak uptake, we defined the end of the dynamic PET acquisition as TTP. Furthermore, the slope of the TAC in the late phase of FET uptake was assessed by fitting a linear regression line to the late phase of the curve (20–50 min post-injection). The slope was expressed as the change of the SUV per hour. This procedure allows for a more objective evaluation of kinetic data compared with an assignment of TACs to earlier reported patterns of FET uptake during dynamic acquisition [33].

The ROIs and VOIs are selected, positioned and validated by two experienced, board-certified specialists in nuclear medicine (G.S., K-J.L.) with more than 10 years of experience in the analysis of FET PET images.

### Neuropathological tumour classification and analysis of molecular markers

All tumours were neuropathologically re-classified according to the WHO Classification of Tumours of the Central Nervous System of 2016 [7]. For molecular biomarker analysis, tumour DNA was extracted from formalin-fixed and paraffin-embedded tissue samples with a histologically estimated tumour cell content of 80% or more. For assessment of the IDH mutation status, the presence of an IDH1-R132H mutation was evaluated by immunohistochemistry using a mutation-specific antibody in a standard immunohistochemical staining procedure as reported before [36, 37]. When immunostaining for IDH1-R132H remained negative, the mutational hot-spots at codon 132 of IDH1 and codon 172 of IDH2 were directly sequenced as reported [10, 38]. The 1p/19q co-deletion status was determined by PCR-based microsatellite analysis as reported [39]. The O^6^-methylguanine-DNA-methyltransferase (MGMT) promoter methylation status was assessed by methylation-specific PCR as described elsewhere [38].

### Survival times

Progression-free survival (PFS) was defined as the time in months between the neuropathologically confirmed glioma diagnosis and tumour progression according to the response assessment in neuro-oncology (RANO) working group [40]. The overall survival (OS) was defined as the time in months between the neuropathologically confirmed glioma diagnosis and death.

### Statistical analyses

Descriptive statistics are provided as mean and standard deviation or median and range. The Student *t* test for independent samples was used to compare two different groups. The Mann-Whitney rank-sum test was used when variables were not normally distributed. The diagnostic performance of FET uptake, as determined by TBR_max_, TBR_mean_, TTP, and slope, was assessed by receiver operating characteristic (ROC) curve analyses using a favourable OS of ≥ 24 months as reference (the median OS in GBM patients is in the range of 15–20 months [41–43], and the 2-year survival rate is 30% [44]; therefore, an OS of ≥ 24 months was considered as favourable). The decision cutoff was considered optimal when the product of paired values for sensitivity and specificity reached its maximum. As a measure of the diagnostic quality of the test, we determined the area under the ROC curve (AUC), its standard error, and the level of significance. Univariate survival analyses were performed using the log-rank test. Multivariate Cox proportional hazards models were constructed to test the relationship between static and dynamic FET PET parameters and other predictors of survival. Parameters that were significant in univariate analyses were included in multivariate models. A *P* value of < 0.05 was considered significant. Statistical analyses were performed using SigmaStat software (SigmaPlot for Windows 11.0, Chicago, IL) and SPSS Statistics software (Release 24.0, SPSS Inc., Chicago, IL, USA).

## Results

### Survival

In the whole cohort (*n* = 60), the median PFS was 12 months (range, 0–75 months), and the median OS was 17 months (range, 0–75 months). In the subgroup of patients with IDH-wildtype gliomas (*n* = 45), the median PFS was 9 months (range, 0–52 months), and the median OS was 15 months (range, 0–52 months). Following biopsy or resection, the vast majority of patients underwent radiotherapy plus an adjuvant treatment option (*n* = 52; 87%) (Table 1).

### Optimal thresholds derived from FET PET parameters

**Fig. 1:**
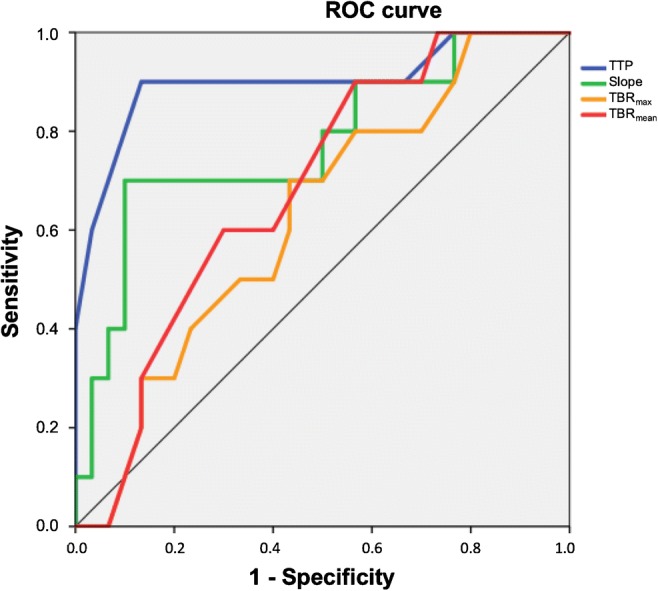
ROC curves for the parameters TTP, slope, TBR_max_, and TBR_mean_

**Fig. 2:**
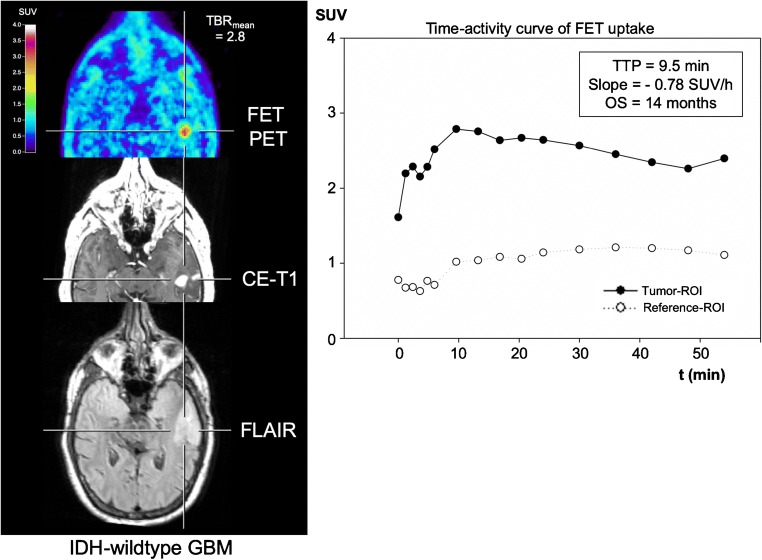
Neuroimages including FET PET, contrast-enhanced MRI, FLAIR-weighted MR image, and the TAC of a patient with an IDH-wildtype GBM and prognostically unfavourable dynamic FET PET parameters (i.e. TTP < 25 min, slope < − 0.103 SUV/h). The OS of that patient was 14 months

**Fig. 3:**
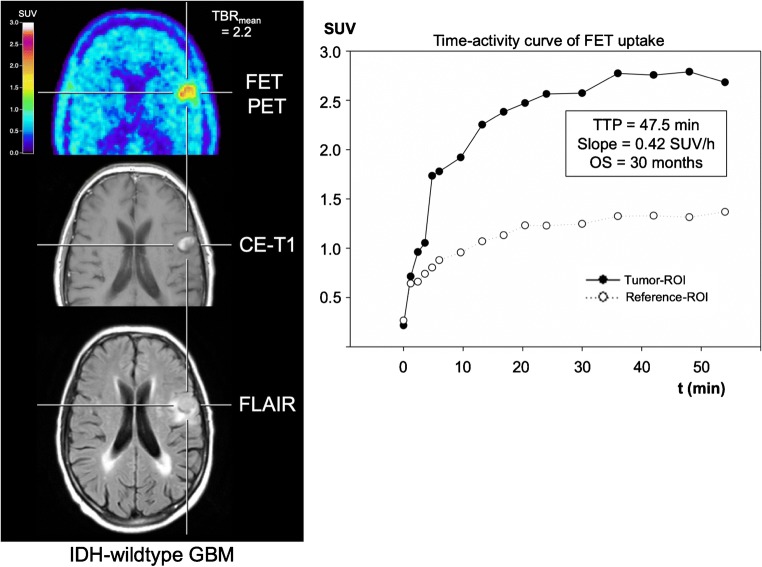
Neuroimages including FET PET, contrast-enhanced MRI, FLAIR-weighted MR image, and the TAC of a patient with an IDH-wildtype GBM and prognostically favourable dynamic FET PET parameters (i.e. TTP > 25 min, slope > − 0.103 SUV/h). The OS of that patient was 30 months

An overview of the results of the optimal thresholds derived from FET PET is provided in Table 2. Specifically, ROC analysis revealed that the dynamic FET PET parameter slope predicts a favourable OS of ≥ 24 months with a sensitivity of 70% and a specificity of 90% (AUC, 0.77 ± 0.09; threshold, − 0.103 SUV/h; *P* = 0.010). The most significant OS prediction of ≥ 24 months could be obtained by the dynamic FET PET parameter TTP (threshold, 25 min; sensitivity 90%; specificity, 87%; AUC, 0.90 ± 0.07; *P* < 0.001) (Figs. 1, 2, and 3). In contrast, the static FET PET parameters TBR_max_, TBR_mean_, and MTV were not prognostic for a favourable OS of ≥ 24 months (AUC, 0.63, 0.69, and 0.56, respectively).

**Table 2:** Diagnostic performance of static and dynamic FET PET parameters

	TBR_max_	TBR_mean_	MTV	TTP	Slope
Threshold	2.55	2.05	11.15 mL	25 min	− 0.103 SUV/h
Sensitivity (%)	70	60	72%	90	70
Specificity (%)	57	70	54%	87	90
AUC ± standard deviation	0.63 ± 0.10	0.69 ± 0.09	0.56 ± 0.09	0.90 ± 0.07	0.77 ± 0.09
*P* value	0.235	0.083	0.56	< 0.001	0.010

*AUC* area under curve from a receiver operating characteristic curve, *MTV* metabolic tumour volume, *TBR*_*max*_ maximum tumour-to-brain ratio of FET uptake, *TBR*_*mean*_ mean tumour-to-brain ratio of FET uptake, *TTP* time-to-peak; *SUV* standardized uptake value

### Univariate survival analysis—influence of general prognostic factors on survival

In the whole cohort (*n* = 60), the univariate survival analysis revealed that glioma patients with IDH-mutant tumours had significantly longer PFS (33 vs. 9 months) and OS (54 vs. 13 months) than patients with IDH-wildtype gliomas (both *P* < 0.001). Similarly, glioma patients with MGMT promoter-methylated tumours had a significantly longer PFS (18 vs. 7 months) and OS (29 vs. 12 months) than glioma patients whose tumours lacked MGMT promoter methylation (both *P* < 0.001). Patients with a KPS of 100% had a significantly longer OS than patients with a KPS between 60 and 90% (29 vs. 13 months; *P* = 0.015). Furthermore, patients with complete resection of the glioma at initial diagnosis had a significantly longer PFS compared with incompletely resected patients or patients who received a biopsy only (13 vs. 10 months; *P* = 0.038), but not a significantly longer OS (*P* = 0.121). Patients aged < 70 years at initial diagnosis did not show longer PFS and OS compared with patients ≥ 70 years (*P* = 0.552 and *P* = 0.108, respectively) (Table 3).

**Table 3:** Results of univariate survival analyses

Factor	Threshold/criterion	PFS		OS	
		*P* value	Survival time	*P* value	Survival time
Age	< 70 vs. > 70 years	0.552	12 vs. 9 months	0.108	22 vs. 17 months
Resection	CR vs. B/PR	0.038	13 vs. 10 months	0.121	23 vs. 15 months
IDH mutation	mutant vs. wildtype	< 0.001	33 vs. 9 months	< 0.001	54 vs. 13 months
MGMT promoter	methylated vs. non-methylated	< 0.001	18 vs. 7 months	< 0.001	29 vs. 12 months
KPS	100% vs. < 100%	0.143	14 vs. 9 months	0.015	29 vs. 13 months
TBR_max_	< 2.55 vs. > 2.55	0.072	12 vs. 7 months	0.004	24 vs. 12 months
TBR_mean_	< 2.05 vs. > 2.05	0.112	14 vs. 7 months	0.038	25 vs. 12 months
TTP	< 25 vs. > 25 min	0.005	13 vs. 7 months	< 0.001	29 vs. 12 months
Slope	> − 0.103 vs. < − 0.103 SUV/h	0.065	9 vs. 6 months	0.021	17 vs. 9 months
MTV	11.15 mL	0.406	12 vs. 7 months	0.048	24 vs. 12 months

*B* biopsy, *CR* complete resection, *IDH* isocitrate dehydrogenase, *KPS* Karnofsky Performance Score, *MGMT*, O^6^-methylguanine-DNA-methyltransferase, *MTV*, metabolic tumour volume, *OS* overall survival, *PFS* progression-free survival, *PR* partial resection, *TBR*_*max*_ maximum tumour-to-brain ratio of FET uptake, *TBR*_*mean*_ mean tumour-to-brain ratio of FET uptake, *SUV* standardized uptake value

### Univariate survival analysis—prediction of survival using static and dynamic FET PET parameters

**Fig. 4:**
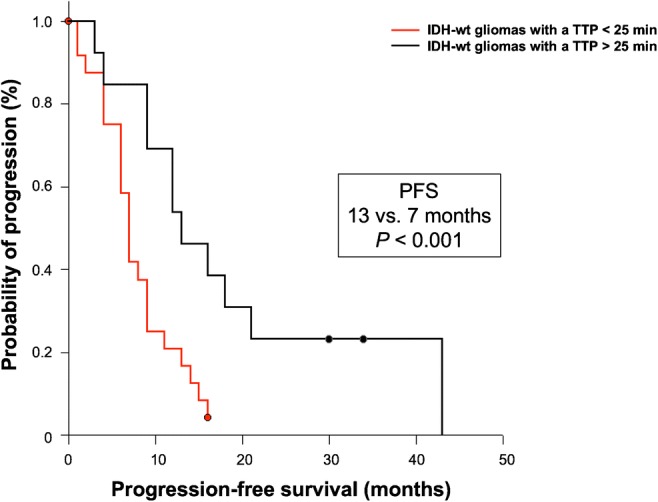
PFS separated by the TTP (threshold, 25 min) within the patient group of newly diagnosed and IDH-wildtype astrocytic glioma of the WHO grades III or IV

**Fig. 5:**
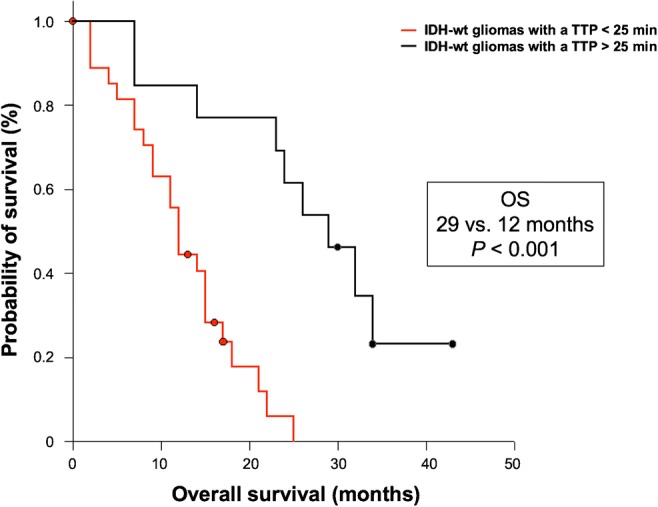
OS separated by the TTP (threshold, 25 min) within the patient group of newly diagnosed and IDH-wildtype astrocytic glioma of the WHO grades III or IV

In patients diagnosed with an IDH-wildtype glioma at initial diagnosis (*n* = 45), the dynamic FET PET parameter TTP (threshold, 25 min) predicted both a significantly longer PFS (13 vs. 7 months) and OS (29 vs. 12 months) (*P* = 0.005 and *P* < 0.001, respectively) (Figs. 4 and 5). Furthermore, the dynamic FET PET parameter slope (threshold, − 0.103 SUV/h) also predicted a significantly longer OS (17 vs. 9 months; *P* = 0.021), but not a significantly longer PFS (*P* = 0.065). Compared with TTP, TBR_max_, TBR_mean_, and MTV were only prognostic for a longer OS at a lower level of significance (*P* = 0.004, *P* = 0.038, and *P* = 0.048, respectively) (Table 3).

### Multivariate survival analysis

Besides MGMT promoter methylation and complete resection at initial diagnosis, the dynamic FET PET parameter TTP remained statistically significant in the multivariate survival analysis (*P* = 0.008; HR, 0.941; 95% CI, 0.900–0.984), indicating an independent predictor for OS. The parameters KPS, TBR_max_, TBR_mean_, slope, and MTV were not statistically significant in the multivariate survival analysis (Table 4).

**Table 4:** Results of multivariate survival analyses in patients with IDH-wildtype glioma

	Threshold	Hazard ratio	95% confidence interval	*P* value
TTP	25 min	0.941	0.900–0.984	0.008
MGMT promoter	methylated	0.337	0.133–0.851	0.021
KPS	100%	1.680	0.752–3.753	0.206
Resection	CR	0.328	0.147–0.730	0.006
TBR_max_	2.55	0.844	0.127–5.620	0.861
TBR_mean_	2.05	0.845	0.033–21.852	0.919
Slope	−0.103 SUV/h	1.199	0.600–2.395	0.607
MTV	11.15 mL	1.017	0.986–1.048	0.294

*CR* Complete resection, *KPS* Karnofsky Performance Score, *MGMT* O^6^-methylguanine-DNA-methyltransferase, *MTV* metabolic tumour volume, *TBR*_*max*_ maximum tumour-to-brain ratio of FET uptake, *TBR*_*mean*_ mean tumour-to-brain ratio of FET uptake, *TTP* time-to-peak, *SUV* standardized uptake value

## Discussion

The main finding of the present study is that dynamic FET PET parameters such as TTP and slope may identify a prognostically more favourable subgroup among patients with newly diagnosed IDH-wildtype astrocytic glioma of the WHO grades III or IV. Our findings suggest that imaging biomarkers derived from dynamic FET PET provide additional prognostic information beyond molecular biomarkers and WHO grades. Thus, FET PET may be valuable for patient counselling and inform treatment planning, thereby allowing stronger emphasis on personalized therapies based on both molecular markers and refined imaging techniques such as dynamic FET PET.

Basically, our findings are line with and extent previous results [18, 26, 27]. Studies in patients with lower-grade and mainly non-enhancing IDH-mutant astrocytomas also highlighted the value of dynamic FET PET parameters (i.e. TAC patterns and TTP) to independently identify patient subgroups with favourable outcome regarding PFS and OS. In comparison to the present study, however, there are differences which warrant discussion.

Like in the present study, Suchorska and colleagues [27] reported in IDH-mutant gliomas of WHO grade II or III an optimal TTP threshold of > 25 min to identify patients with favourable outcome. Using a more stringent TTP threshold of > 12.5 min, the identification of IDH-wildtype glioma patients of WHO grade II or III (*n* = 76) with favourable outcome was, however, not possible. Conversely, a recent study reported that the TTP threshold of 12.5 min allows the distinction between two prognostic subgroups, including lower-grade IDH-wildtype glioma patients (*n* = 27) [27]. We here demonstrate that the identification of two distinct prognostic subgroups among patients with IDH-wildtype astrocytic gliomas of WHO grades III or IV is possible using a TTP threshold of 25 min. As suggested previously [45], one reason might be the slightly different dynamic FET PET imaging protocol used in different centres, e.g. scanning time of 50 min in our centre compared with 40 min in other centres [18, 26, 27]. Furthermore, differences in WHO grades (i.e. a higher proportion of IDH-wildtype gliomas of WHO grade III or IV in the present study) may impact on the TTP threshold.

As reported for several other indications [15–22], TAC patterns (e.g. increasing/decreasing TACs), either combined with TTP [18] or alone [26], also seem to have prognostic significance for glioma subgroups. Notwithstanding, the interpretation of TAC patterns is subjective to a certain degree. To provide a more objective and reader-independent characterization of TAC patterns in the present study, we calculated the slope by fitting a linear regression line to the late phase of the TAC. Importantly, similar to TTP, albeit at a lower level of significance, the slope value also allows identifying patients with an improved OS within neuropathologically defined glioma subgroups.

The following limitations of our study need to be discussed. The study is based on retrospective data, and the results need to be confirmed in a prospective study. Another putative weakness is the relatively small number of patients. Nevertheless, compared with other studies [18, 26], our dataset includes mostly patients with unfavourable IDH-wildtype astrocytic glioma of WHO grades III and IV, allowing a more profound prognostic evaluation within this group of patients. Furthermore, from a practical point of view, the acquisition and post-processing of a dynamic FET PET scan is more complex and time-consuming than the evaluation of TBRs, which are commonly used in clinical routine, and therefore hampers a widespread use of this powerful imaging technique. In addition, one might argue that PET imaging protocols are not sufficiently standardized for widespread use on a routine basis. Notwithstanding, in 2019, major European and American medical societies for nuclear medicine and neuro-oncology (i.e. the SNMMI (Society of Nuclear Medicine and Molecular Imaging), EANM (European Association of Nuclear Medicine), EANO (European Association of Neuro-Oncology), and the RANO group) have published joint practice guidelines for static and dynamic PET imaging [30] which may help to harmonize acquisition protocols and post-processing in the near future.

Another important point which needs to be discussed is the detection of the most malignant parts for neuropathological evaluation. In the present study, tissue was obtained from tumour parts with the highest metabolic activity on FET PET. It has been demonstrated that this procedure identifies the most malignant tumour parts in heterogeneous gliomas with high accuracy and therefore helps to avoid undergrading [17]. Furthermore, in gliomas with an IDH mutation, mutant IDH proteins are ubiquitously expressed in tumour cells, suggesting that IDH mutations are an early causative event in the genesis of these brain tumours [46]. This also applies to the MGMT methylation status [47], and other molecular markers such as 1p/19q co-deletion and TP53 [48]. Thus, due to a FET PET-guided tissue removal of the tumour parts with the highest metabolic activity and the homogenous distribution of various molecular markers within gliomas, a histomolecular misclassification seems to be unlikely.

In conclusion, our data suggest that within a histomolecularly defined subgroup of patients with newly diagnosed IDH-wildtype GBM and AA, dynamic FET PET allows the identification of patients with improved OS. Especially in the subgroup of patients with IDH-wildtype tumours and reduced clinical condition (e.g. older patients with a reduced KPS), our FET PET findings may be helpful for patient counselling, i.e. to decide whether an aggressive treatment regimen including radio- and chemotherapy with an increased risk of severe side effects should be administered or not. Accordingly, prospective data are warranted to further improve the level of confidence.
